# Genetic variation in morphological traits in cotton and their roles in increasing phosphorus-use-efficiency in response to low phosphorus availability

**DOI:** 10.3389/fpls.2022.1051080

**Published:** 2022-11-30

**Authors:** Mirezhatijiang Kayoumu, Xiaotong Li, Asif Iqbal, Xiangru Wang, Huiping Gui, Qian Qi, Sijia Ruan, Ruishi Guo, Qiang Dong, Xiling Zhang, Meizhen Song

**Affiliations:** ^1^ Institute of Cotton Research of Chinese Academy of Agricultural Sciences/Zhengzhou Research Base, State Key Laboratory of Cotton Biology/School of Agricultural Sciences, Zhengzhou University, Anyang, Henan, China; ^2^ Western Agricultural Research Center of Chinese Academy of Agricultural Sciences, Changji, China

**Keywords:** genetic variation, cotton genotypes, morphological traits, plasticity, phosphorus use efficiency

## Abstract

Phosphorus (P) is an essential macronutrient required for fundamental processes in plants. Trait plasticity is crucial for plant adaptation to environmental change. Variations in traits underlie diverse phosphorus (P) acquisition strategies among plants. Nevertheless, how the intraspecific plasticity and integration of morphological traits contribute to Phosphorus-Use-Efficiency (PUE) in cotton is unknown. In this study, 25 morphological traits were evaluated in 384 cotton genotypes grown with low P (LP, 10μmol. L^−1^) and normal nutrition (CK, 500μmol. L^−1^) to assess the genetic variability of morphological traits and their relationship to phosphorus use efficiency. Results revealed a large genetic variation in mostly morphological traits under low P. Significant enhancement in root traits and phosphorus efficiency-related traits like PUE was observed at LP as compared to CK conditions. In response to low P availability, cotton genotypes showed large plasticity in shoot and total dry biomass, phosphorus, and nitrogen efficiency-related traits (i.e., phosphorus/nitrogen use efficiency, phosphorus/nitrogen uptake efficiency), and most root traits, but a limited response in root dry biomass, taproot length, root surface area, root volume, and SPAD value. In addition, significant correlations were observed between PUtE (phosphorus uptake efficiency), NUE (nitrogen use efficiency), TDB (total dry biomass), and RTD (root tissue density) with PUE under both P supply level and phosphorus stress index, which may be a key indicator for improving PUE under LP conditions. Most root traits are most affected by genotypes than nutrition level. Conserved PUE is more affected by the nutrition level than the genotype effect. Principal component analysis depicted the comprehensive indicators under two P supply conditions were mainly reflected in root-related traits and morphological indicators such as dry matter biomass. These results indicate that interspecific variations exist within these cotton genotypes and traits. Our study provides suggestions for future research to enhance the ability of the earth system model to predict how crops respond to environmental interference and provide target quality for cotton breeding in phosphorus-deficient areas.

## Introduction

As a component of vital macromolecules including nucleic acids, nucleotides, and phospholipids 43 as well as having major metabolic and protein-regulating functions in the form of phosphate (Pi) or Pi 44 esters, phosphorus (P) is one of the most important essential nutrients (Paz-Ares et al., 2022). Plants absorb only 15% to 25% of the supply of P fertilizer. The remainder is released into the environment, where it causes soil erosion and water eutrophication, resulting in low P availability in soil. (Conley et al., 2009; Johnston et al., 2014).

Plants have various adaptive strategies for boosting P absorption and mobilization to deal with reduced P availability in soil (Guo et al., 2022a). Plants can increase P uptake by enhancing their P acquisition efficiency or phosphorus use efficiency (PUE) (Hammond et al., 2009; White et al., 2013; Dissanayaka et al., 2018). P efficiency is primarily determined by two factors: the ability to obtain P from soils through root characteristics and underground activities (Elanchezhian et al., 2015; Wang and Lambers, 2019) and P utilization, which describes cellular processes including the remobilization of P (Vance, 2003; Wang et al., 2010). As a response, it is becoming increasingly clear to cultivate P-efficient crop cultivars. It is required to boost their PUE and P acquisition, improving P balance in agroecosystems (Rose and Wissuwa, 2012; Cong et al., 2020; Watanabe et al., 2020). An increasing amount of research has been done over the past 20 years to improve plant PUE (Paz-Ares et al., 2022). These studies have primarily been devoted to analyzing the processes that govern the P starvation (PS) rescue system (PSR), which gives plants the capacity to adapt their growth to low-P situations (Paz-Ares et al., 2022). A series of plant adaptations are triggered by P deficiency to reduce P consumption and enhance P absorption and recycling (Lynch, 2019). Such adaptive response techniques typically cause internal molecular alterations and external morphological plasticity in crops (Guo et al., 2022b). Root and shoot architecture changes due to the typical morphological plasticity in response to low P stress (Niu et al., 2013). Under P-deficient stress, roots often grow longer and denser to increase the area of contact between the roots and soil and improve P uptake (Peret et al., 2011; Mori et al., 2016).

Mainly the P acquisition features have been extensively utilized to increase the PUE of crops (Wissuwa et al., 2008). But it’s essential to investigate the characteristics that control PUE. The main focus in the last few decades has been on improving PUE, which is linked to root and shoot system architecture and rhizosphere processes and has been extensively researched (Lynch, 2011; Pang et al., 2018; Zhang et al., 2018; Lynch, 2019). Neumann and Martinoia (2002) proved lateral root growth was preferred over primary root growth in moderate P concentrations. In Arabidopsis, the root adaptive response induced by low P increases root P uptake capacity by altering root structure and promoting the expression of multiple PUE-related genes. (Yang et al., 2021). Additionally, it was found in traditional rice (Oryza sativa) that cultivars with longer roots and early root development controlled by the protein kinase PSTOL1 may affect plant phosphorus uptake, thereby enhancing phosphorus acquisition (Sun et al., 2014). In addition, phosphorus use efficiency can be significantly affected by the size of plant P pools and the proportion of phosphorus allocated to and remobilized from collections and tissues throughout the plant growth cycle (Sandaña, 2016). Sandaña and Pinochet (2014) indicated that more giant root-to-shoot ratio corn hybrids might be created to increase PUE. Genotypic variation in PUE and its components has been assessed in several crops. A study on wheat showed that the P-efficient cultivar maintained higher inorganic P concentrations in its organs than the P-inefficient cultivar (Aziz et al., 2014). Rose et al. (2015) found that under various P supply conditions, compared with the P-inefficient rice genotypes, the P-efficient rice genotypes may increase their PUE and preserve essential functions by modifying the root architecture brought on by P starvation. For particular, late-maturing source groups from tropical maize landraces with high net P uptake, a high dry matter and P partition to the grain, and the maintenance of high grain P concentrations may be used as components for characteristics of adaptive value for P-limited situations (Pandey et al., 2014).

Cotton (Gossypium hirsutum L.) is a vital crop for the global economy that produces natural fiber, the primary raw material for the textile industry (Zhang et al., 2021). Cotton agronomic properties are significantly impacted by P deficit, which also lowers plant height, leaf area, and dry matter quality (Li et al., 2020). However, few studies examined PUE and the significance of PUtE in a wide variety of cotton genotypes in response to various P availabilities. Given the limitations outlined above and the lack of knowledge regarding the genotypic variance of PUE in cotton. The specific aims of this work were to (1) estimate the genetic variability of morphological traits’ response to changes in P availability, (2) investigate the range of plasticity of each trait, and (3) evaluate their contribution to increasing PUE and its components under low P availability conditions.

## Materials and methods

### Plant material

The research was set up in the growth chamber at the Institute of Cotton Research, the Chinese Academy of Agricultural Sciences, Anyang, China. The 384 cotton genotypes used in this experiment are based on a breed population determined in previous studies (not published). The breeding years information is shown in (**Supplementary Table 1**).

### Experiment design

384 cotton genotypes seeds were incubated for 7 days in a sand and vermiculite combination in a growth chamber. The uniform seedlings were transplanted into 8L plastic boxes after germination. The plants were grown under natural light in a greenhouse. 28/20°C (day/night) temperatures, 60% humidity levels. At the initial stage, the plants were provided with 100 mL dH_2_O every other day, and seedlings were supplied with 1/2-strength, followed by a full-strength-Hoagland solution. Every week, each plant was randomly to counteract the impact of position (Liang et al., 2021). A total of two treatments were set: normal nutrition (CK) and low P (LP). Four plants were used per replicate. Four replicates for each genotype. Seedlings were treated with various nutrient supplies at the two true leaves stage. The conventional nutrient solution is as follows: (0.1mmol/L EDTA·Fe·Na,1mmol/L MgSO_4_·7H_2_O, 2μmol/L ZnSO_4_·7H_2_O, 46μmol/L H_3_BO_3_, 4μmol/L MnCl_2_·4H_2_O, 0.3μmol/L CuSO_4_·5H_2_O, 0.12μmol/L (NH_4_)_6_Mo_7_O_24_·4H_2_O, 0.5mmol/L KH_2_PO_4_, 2.5mmol/L Ca (NO_3_)_2_·4H_2_O, pH 5.5) containing 10 (LP), 500 (CK) μM KH_2_PO_4._ Every week new solutions were added, and an electric pump aerated them. Plants started to show clear signs of P treatment after four weeks. Then various morphological characteristics were measured.

### Plant morphological characteristics

The dry biomass of the shoots and roots was then calculated after 48 hours of drying at 72°C. Roots were dipped in 0.1% (w/v) toluidine blue for 5min and then scanned at 300 dpi using a desktop scanner (Epson Perfection 11000xL, Long Beach, CA, USA). Afterward, root images were loaded into a WinRHIZO analysis system (version 2012B, Regent Instruments Canada, Montreal, Canada). The root traits were determined according to the established method (Wen et al., 2019). A portable chlorophyll instrument for measuring the relative chlorophyll content (SPAD 502 Meter, Minolta Corporation, Tokyo, Japan). Based on the above measurements, the description and algorithm of phenotypic traits of the root system are shown in (**Table 1**).

**Table 1:** Description of 25 measured morphological traits in 384 cotton genotypes.

Traits	Abbreviation	Description	Units
Taproot length	TRL	Length from rhizome separation to the farthest point of the main root	cm
Total root length	TRH	The total length of all roots per plant	cm
Root volume	RVE	The total volume of all roots per plant	cm^3^
Root average diameter	RAD	The average diameter of all roots (including lateral roots) per plant	mm
Root surface area	RSA	Total root surface area of roots per plant	cm^2^
Root tips number	RTN	Average tips number of all roots per plant	Num
Root tissue density	RTD	Total root dry mass divided by root volume	g·cm^-3^
Specific root tips density	SRTD	Average root tips number divided by root dry mass	g^-1^
Specific root length	SRL	Total root length divided by root dry mass	cm·g^-1^
Specific root area	SRA	Root surface area divided by root dry biomass	cm^2^·g^-1^
SPAD Value	SPAD	The relative value of chlorophyll content in leaves	Num
Shoot fresh weight	SFW	The weight measured immediately after the fresh shoots are collected	g·plant^-1^
Root fresh weight	RFW	The weight measured immediately after the fresh roots are collected	g·plant^-1^
Shoot dry biomass	SDB	Total shoot dry mass per plant	g·plant^-1^
Root dry biomass	RDB	Total dry mass of all roots per plant	g·plant^-1^
Total dry biomass	TDB	constant whole plant weight	g·plant^-1^
Root to shoot ratio	R/S	The ratio of the dry matter weight of the aboveground part and the underground part	%
Total N concentration	TNC	N concentration in plant aboveground and underground tissues	mg/g
Total N accumulation	TNA	Average N content per unit weight of seedlings	g/g·
N use efficiency	NUE	Weight of dry matter produced by unit N absorption	g^2^/mg^-1^
N uptake efficiency	NUtE	Total N uptake of plants	mg·g^-1^
Total P concentration	TPC	P concentration in total plant	mg/g
Total P accumulation	TPA	Average P content per unit weight of seedlings	g/g·
P use efficiency	PUE	Weight of dry matter produced by a unit phosphorus absorption	g^2^/mg^-1^
P uptake efficiency	PUtE	Total phosphorus uptake of plants	mg·g^-1^

### Measurements P and N efficiency traits

After being ground into a fine powder, the dried samples of shoot and root were weighed at about 0.12 g each. The nitrogen and phosphorus concentrations were measured using the Bran ^+^ Luebbe Continuous-Flow AutoAnalyzer III (AA3-Germany) after 3 hours of digestion with H_2_SO_4_-H_2_O_2_. PUE and NUE definitions for cotton genotypes grown at various phosphorus concentrations are also included and were calculated as Total P and N accumulation (TPA, TNA) = the P and N concentration multiplied by total plant dry matter, P and N uptake efficiency (PutE, NutE) = the total plant dry matter divided by P and N concentration, P and N use efficiency (PUE, NUE) = TPA, TNA divided by root dry matter, follows by (Iqbal et al., 2019) with minor modifications.

### Statistical analysis of evaluated data

All data were analyzed for normality (Kolmogorov–Smirnov test) and tested for homogeneity of variance (Leven median test). The Global ANOVA was used to evaluate the data (genotype and nutrition level as main factors), and the means were separated by Tukey’s honest significant difference (HSD) test (P < 0.05). The standard deviation to a collection means ratio was used to estimate the coefficient of variation for each parameter.

The term “phenotypic plasticity” (PL) refers to the alteration in phenotype brought on by environmental variance (Bradshaw, 1965; Tan et al., 2020). This is the variance resulting from the interaction between genotype and environment: $\sigma^{2}\times +\sigma^{2}{\text{E}}_{\text{G}\times \text{E}}$ . On the other hand, plasticity (PL) is a quote of phenotypic variance ( $\sigma^{2}{\;}_{\text{P}}$ ), calculated as $\sigma^{2}\text{pl}=\sigma^{2}{\;}_{\text{PL}}/\sigma^{2}{\;}_{\text{P}}$ (Auld et al., 2010). Finally, the heritable plastic variation caused by the interaction of genetics and environment (h^2^PL) was estimated as $\sigma^{2}\text{G}\times_{\text{E}}/\sigma^{2}{\;}_{\text{P}}$ . Based analysis of variance (ANOVA) model: Phenotype = Genotype + nutrition level + Genotype by nutrition Level ^+^ Residuals, where nutrition level was treated as a fixed effect, and Genotype and Genotype‐by‐nutrition level were treated as random effects. Conversely, trait broad sense heritability (h^2^B) was calculated as $\sigma^{2}{\;}_{\text{G}}/\sigma^{2}{\;}_{\text{P}}$ , where $\sigma^{2}{\;}_{\text{G}}$ is the genetic variance component (attributable to variation among genotypes), while $\sigma^{2}{\;}_{\text{P}}$ is the total phenotypic variance, as previously defined.

Principal component analysis (PCA) was calculated in OriginPro (2021) (OriginLab Corporation, Northampton, MA, USA). Morphophysiological traits were used to calculate the correlation relationships in Microsoft excel. The figures were drawn with OriginPro (2021). All the data results are expressed as the mean of four replications. Biplot graphs for each characteristic displayed the average values for each accession obtained at LP (plotted along the vertical axis) compared to those at CK (plotted along the horizontal axis) levels. We could determine which attribute was most significantly impacted by the P supply due to the biplot analysis. The distribution of genotypes along the diagonal bisector of biplots explained their genetic variation. The distance from the bisector revealed each genotype’s ability to adapt to the P availability.

## Results

### Effects of P availability on the growth of cotton seedlings

**Figure 1:**
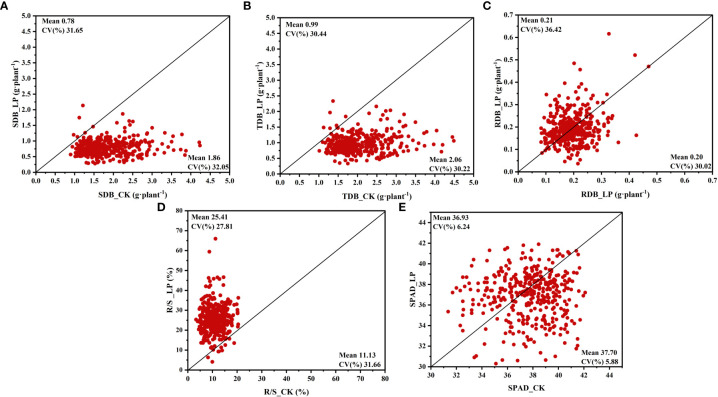
Variation in shoot and root fresh and dry weight and root shoot ratio of 384 cotton plants. **(A)** shoot dry biomass (SDB), **(B)** total dry biomass (TDB), **(C)** root dry biomass (RDB), **(D)** root shoot ratio (R/S), **(E)** SPAD value (SPAD). The horizontal line in the picture is normal nutrition conditions (CK), and the vertical line is low phosphorus (LP).

Cotton genotypes’ responses to the two P treatments were significantly different in morphological characteristics (**Figure 1**, **Supplementary Table S2**). In this study, The growth of the cotton genotypes under LP conditions was markedly slower than under CK conditions (**Supplementary Figure S1**). Compared with CK conditions, shoot dry biomass (SDB) and total dry biomass (TDB) in LP conditions decreased by 57.86% and 51.95%, respectively (**Figures 1A, B**, **Supplementary Table S2**). In contrast, root dry biomass (RDB) exposed to LP conditions was stimulated by 5.93% (**Figure 1C**, **Supplementary Table S2**). Similarly, the root-to-shoot ratio (R/S) was significantly increased by 128.34% under the LP condition (**Figure 1D**, **Supplementary Table S2**). However, SPAD values ​​were not significantly different at the two P supply levels (**Figure 1E**, **Supplementary Table S2**).

Under the LP condition, TDB and RDB showed higher coefficients of variation (CV) of 0.32 and 0.36, respectively, compared to CK conditions. A similar trend was observed for SFW and RFW (**Supplementary Table S2**). In contrast, compared to LP conditions, SDB had a higher CV under CK conditions (**Figure 1A**). The R/S in the CK condition showed a remarkably higher CV (0.32 vs 0.28) compared to the LP condition (**Figure 1D**). Interestingly, the CV of SPAD values ​​under CK and LP conditions were 0.06 and 0.06, respectively, the lowest among the above traits (**Figure 1E**).

Among the relative values of the above traits, the highest CV is R/S, indicating that the R/S of each cotton genotype is more susceptible to the influence of P supply and has a higher variation (**Supplementary Table S2**). The lowest CV was the SPAD value, indicating that the SPAD value was least affected by P availability (**Supplementary Table S2**). The considerable variability in R/S prompted further research into other root morphological traits (**Supplementary Table S2**).

### Responses of root morphological traits to P supply

**Figure 2:**
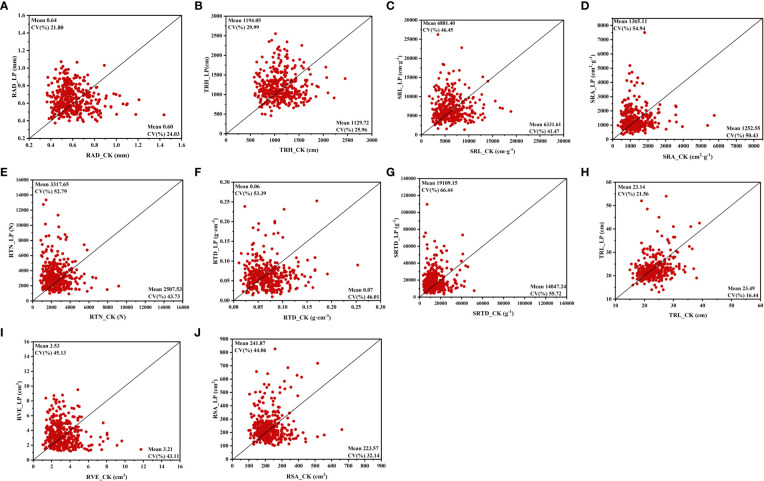
Variation in root morphological trait of 384 cotton plant accessions exposed to different Pi supplies for 28 days. **(A)** root average diameter (RAD), **(B)** total root length (TRH), **(C)** specific root length (SRL), **(D)** specific root area (SRA), **(E)** root tips number (RTN), **(F)** root tissue density (RTD), **(G)** specific root tips density (SRTD), **(H)** tap root length (TRL), **(I)** root volume (RVE), **(J)** root surface area (RSA). The horizontal line in the picture is normal nutrition conditions (CK), and the vertical line is low phosphorus (LP).

Our results indicated that the availability of phosphorus significantly impacted the root morphological traits of cotton genotypes. Mainly all root morphological traits reduced as the concentration of phosphorus increased; however, the degree of increase varied by genotype (**Figure 2**; **Supplementary Table S2**). In this research, the mean root diameter (RAD) and total root length (TRH) of the LPconditions were significantly increased by 7.92% and 5.69%, respectively (**Supplementary Table S2**). Similarly, the LP conditions specific root length (SRL), root surface area (RSA), and specific root area (SRA) increased by 8.68%,8.19%, and 8.99%, respectively (**Figures 2C, D, J**, **Supplementary Table S2**). In addition, LP conditions can stimulate a significant increase in root tip number (RTN), root tissue density (RTD), and specific root tip density (SRTD) (**Figures 2E-G**, **Supplementary Table S2**). Unlike the rise of other root characteristics under LP conditions, tap root length (TRL) and root volume (RVE) were less affected by LP conditions, with no significant difference between the two P supply levels (**Figures 2H, I**).

Different P supplies also affected the CV of each morphological trait of the root system. The results showed that the CV of each trait decreased with the increase in P supply (**Figure 2**). Under CK conditions, SRA (0.50 CV) and SRTD (0.56 CV) had higher variability than other root traits. Meanwhile, these two traits were more sensitive to LP conditions, and the CV is 0.55 and 0.66, respectively (**Figures 2D, G**). Among the CV of the relative values ​​of each trait, CV was the highest in RTD, RTN, and SRTD and lowest in TRL, which is consistent with the degree of variation of each root morphological trait at the two P supply levels (**Supplementary Table S2**). The results showed that P availability substantially impacted cotton root morphological traits.

### Responses of P and N efficiency indexes to P availability

**Figure 3:**
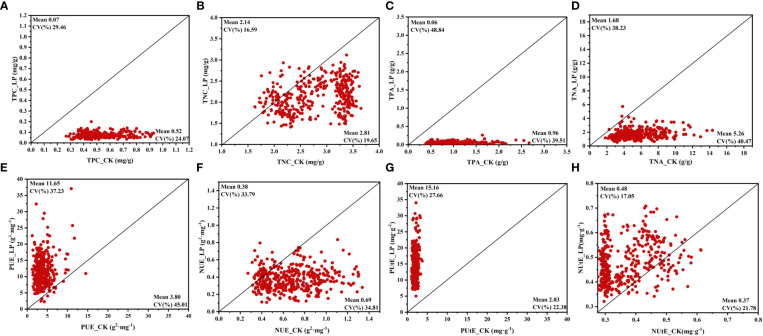
Variation in phosphorus and nitrogen efficiency traits of 384 cotton plant accessions exposed to different P supplies for 28 days. **(A)** total phosphorus concentration (TPC), **(B)** total nitrogen concentration (TNC), **(C)** total phosphorus accumulation (TPA), **(D)** total nitrogen accumulation (TNA), **(E)** phosphorus use efficiency (PUE), **(F)** nitrogen use efficiency (NUE), **(G)** phosphorus uptake efficiency (PUtE), **(H)** nitrogen uptake efficiency (NUtE). The horizontal line in the picture is normal nutrition conditions (CK), and the vertical line is low phosphorus (LP).

To determine the potential of cotton genotypes, the P and N accumulation, P and N use efficiency, and P and N uptake efficiency were recorded. Various P concentrations greatly influence the PUE and PUtE in cotton plants. In this study, compared CK conditions, the TPC and TNC in LP conditions were decreased by 86.32% and 23.88%, respectively (**Figures 3A, B**; **Supplementary Table S2**). Similarly, the TPA and TNA of the cotton genotype under the LP condition were significantly reduced by 94.11% and 68.06%, respectively (**Figures 3C, D**, **Supplementary Table S2**). Among these traits, compared with the CK conditions, the PUE and PUtE under LP conditions showed the highest response to P supply, which were increased by 206.47% and 648.05% in the LP condition, respectively (**Figures 3E, G**; **Supplementary Table S2**). Similarly, the NUtE under LP conditions increased by 29.44% (**Figure 3H**; **Supplementary Table S2**). In contrast, the NUE decreased by 45.16% under LP conditions compared to CK conditions (**Figure 3F**; **Supplementary Table S2**).

Among these phosphorus and nitrogen efficiency traits, the TNA and TPA under LP conditions had the highest CV of 0.38 and 0.49, respectively (**Figures 3C, D**). In addition, the PUE CV value in the CK condition was 0.45, which was significantly higher than the other above traits (**Supplementary Table S2**). The CV for TNC was low, at 0.20 and 0.17 under CK and LP conditions, respectively, also highlighting the similar distributions along the bisector (**Figure 3**). In addition, the CV of PUtE was not significantly different between the two P supply levels, being 0.22 and 0.28 in LP and CK conditions, respectively. A similar trend was observed for NUtE (**Figures 3G, H**). Among the relative values ​​for these traits, the highest CV trait was TPA of 0.56, and the lowest trait was NUtE of 0.23 (**Supplementary Table S2**). Except for TNC and NUtE, the remaining traits had significant genotypic variation at both P supply levels (**Figure 3**).

### Global ANOVA, plasticity, genetic variation, heritability

**Figure 4:**
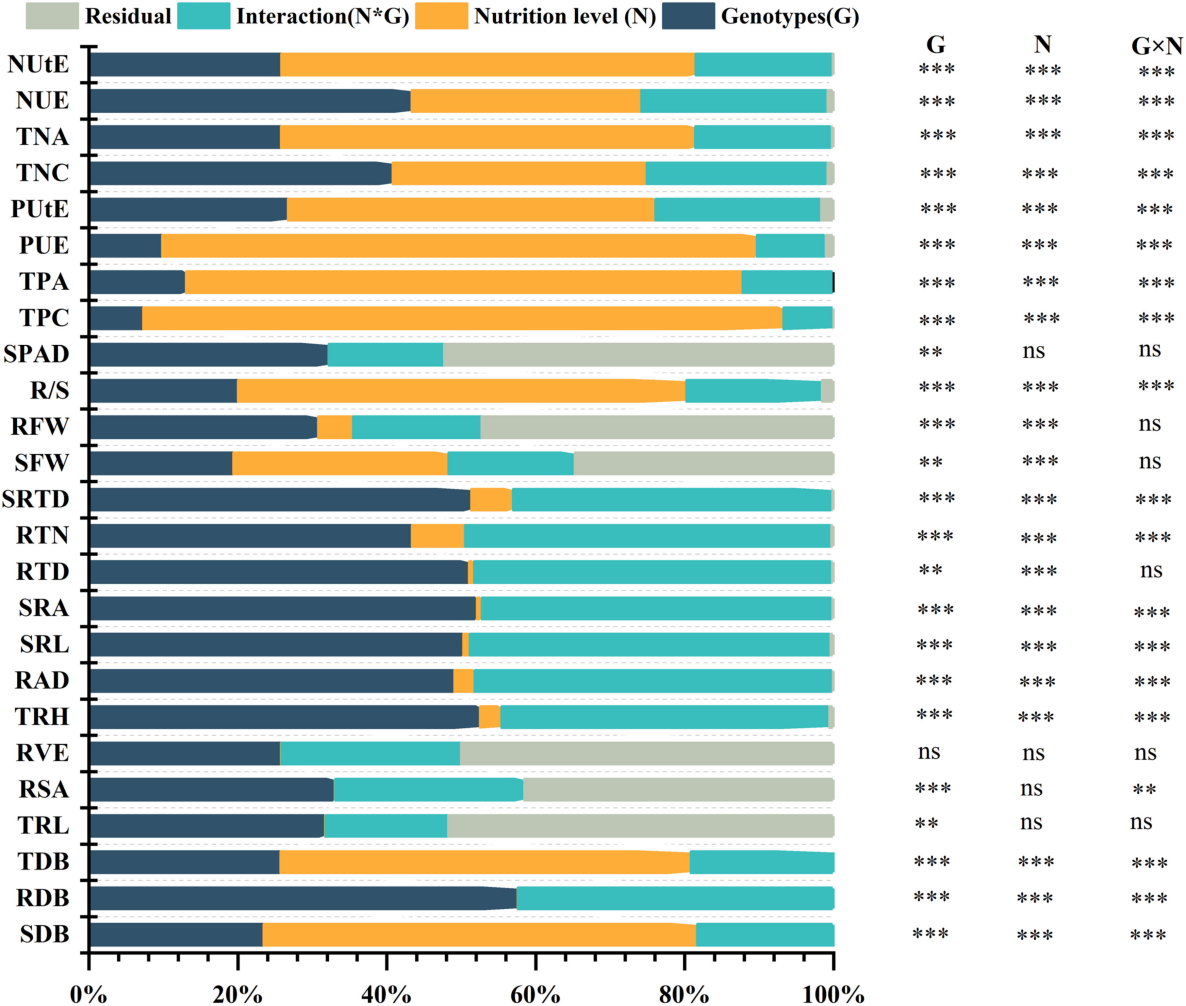
Global ANOVA of morphological traits of 384 cotton genotypes in response to different P supplies. The main effects (Genotypes (G) and nutrition level (N), interaction (G×N), and residual (R) represent as a percentage of type III sums of squares. P-values of the F-test are indicated. **: P < 0.01; ***: P < 0.001; ns: not significant.

**Figure 5:**
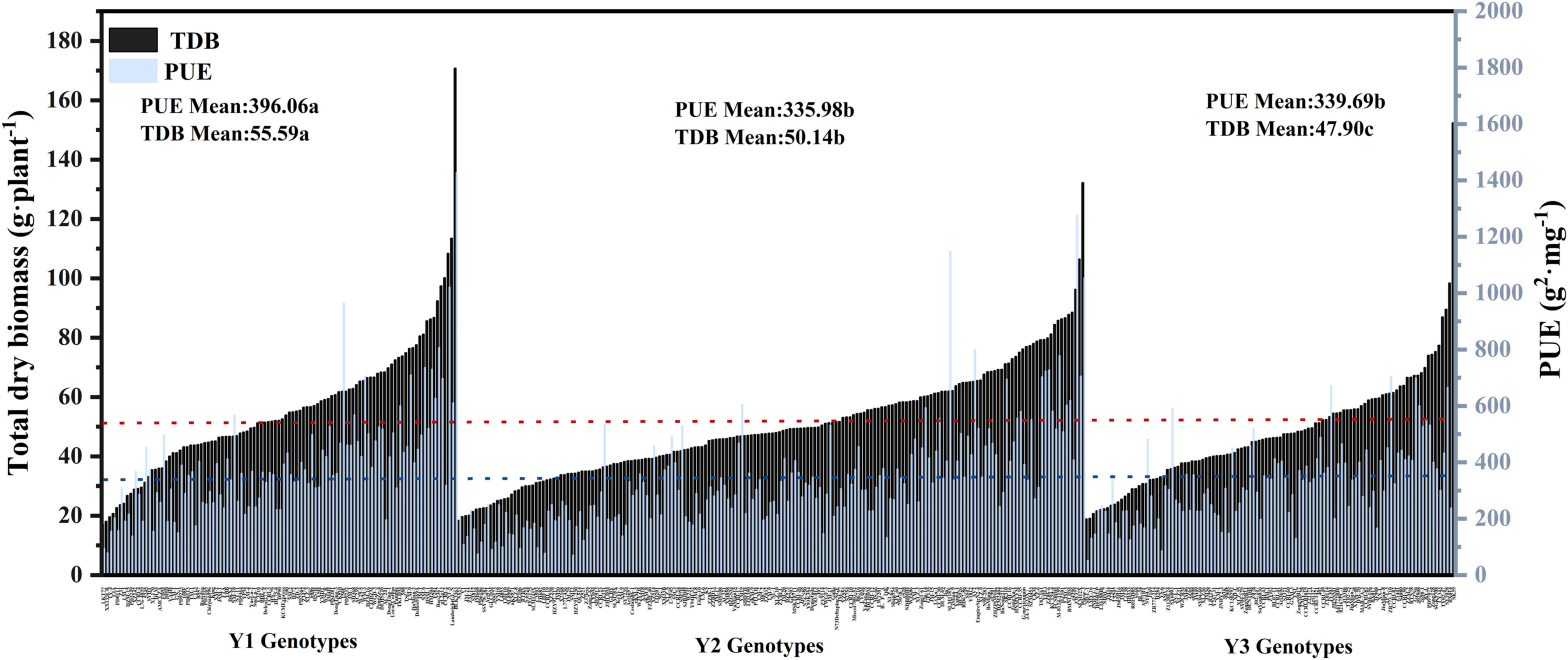
Genotypic variation in PUE and TDB relative value of 384 cotton genotypes grown in a hydroponic phenotyping platform. Mean values were plotted by three groups of genotypes: 101 Y1 genotypes, 177 Y2 genotypes, and 105 Y3 genotypes. (Y1: breeding years before 1980, 101 varieties in total, no chemical fertilizer stage of cotton production.Y2:breeding years: 1981-2000, 177 varieties in total, cotton production (small amount of chemical fertilizer stage).Y3: from 2001 to now, there are 105 varieties in total, and cotton production is at the stage of excessive chemical fertilizer). The mean values of PUE for each genotype group (colored bars) and all genotypes (blue dashed line) are presented. The mean values of TDB for each genotype group (colored bars) and all genotypes (red dashed line) are shown.

Global ANOVA was used to analyze all features within the various nutrition supplies categories to establish the proportion of explained variance owing to genetics, environment, and their interaction (**Figure 4**). This study examined 25 morphological and physiological traits in total. Each attribute that was found had a significant impact on genotypes (G), N (Nutrition level), and their interaction (G×N). In each of these parameters, there were strong G×N interactions (**Figure 4**). All of the characteristics had substantial genetic effects, which accounted for 7% to 58% of the variation. Several root attributes (e.g., SRTD, RTN, RTD, SRA, SRL, RAD, and TRH), over 40% of the total variance was caused by genetic effects (**Figure 4**; **Supplementary Table S3**). In addition, PUE and TDB are also strongly affected by the genotype effect, with apparent genotype variation (**Figure 5**). In addition, the discovered traits were also highly impacted by nutrition level, and nutrition level effects were 1% to 86% of the overall variance among the traits, particularly by phosphorus and nitrogen efficiency traits attributes (e.g., NUtE, TNA, PUE, PUtE, TPA, TPC), meanwhile it also significantly affected SDB and TDB (**Figure 4**, **Supplementary Table S3**).

**Figure 6:**
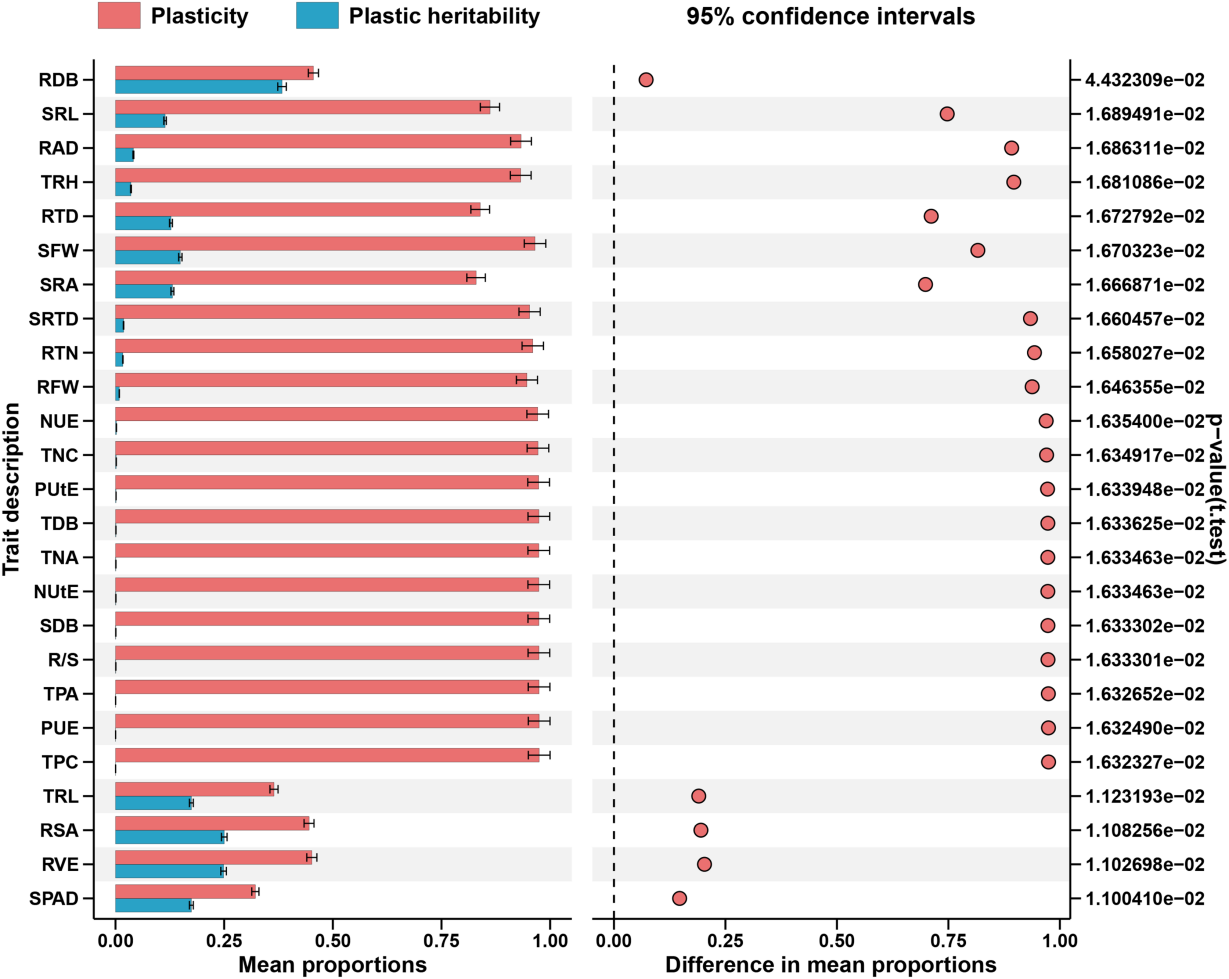
Absolute plasticity of the measured traits in response to both P supply. Trait notations: shoot dry biomass (SDB), root dry biomass (RDB), total dry biomass (TDB), taproot length (TRL), root surface area (RSA), root volume (RVE), total root length(TRH), root average diameter (RAD), specific root length (SRL), specific root area (SRA), root tissue density (RTD), root tips number (RTN), specific root tips density (SRTD), shoot fresh weight (SFW), root fresh weight (RFW), root shoot ratio (R/S), SPAD value (SPAD), total phosphorus concentration (TPC), total phosphorus accumulation (TPA), phosphorus use efficiency (PUE), phosphorus uptake efficiency (PUtE), total nitrogen concentration (TNC), total nitrogen accumulation (TNA), nitrogen use efficiency (NUE), nitrogen uptake efficiency (NUtE). Error bars are standard errors of the mean (n = 384).

Almost all the examined traits had significant impacts by the (G×N) interactions, accounting for 2%–48% of the total variance. They played a substantial part in the total variance (**Figure 4**; **Supplementary Table S3**). The cotton genotypes demonstrated considerable plasticity (PL) for several morphological traits, extending from 0.3300 to 0.998 (**Supplementary Table S4**). TPA, PUE, and TPC exhibited the highest PL values, while the lowest ones were observed in SPAD Value, followed by TRL and RSA (**Figure 6**). Conversely, broad sense h^2^B ranged from 0.69 to 1.00 and 0.70 to 1.000 at LP and CK, respectively (**Supplementary Table S4**). At LP conditions, the highest values of h^2^B were detected in RAD and RDB (**Figure 6**). By contrast, SRA, and RTD, exhibited the lowest ones under LP conditions (**Figure 5**). Similarly, under the control conditions, the h^2^B values of SRA and RTD were also the lowest, while RAD showed the highest h^2^B (**Figure 6**).

### Correlation among morphological traits and associated with PUE

**Figure 7:**
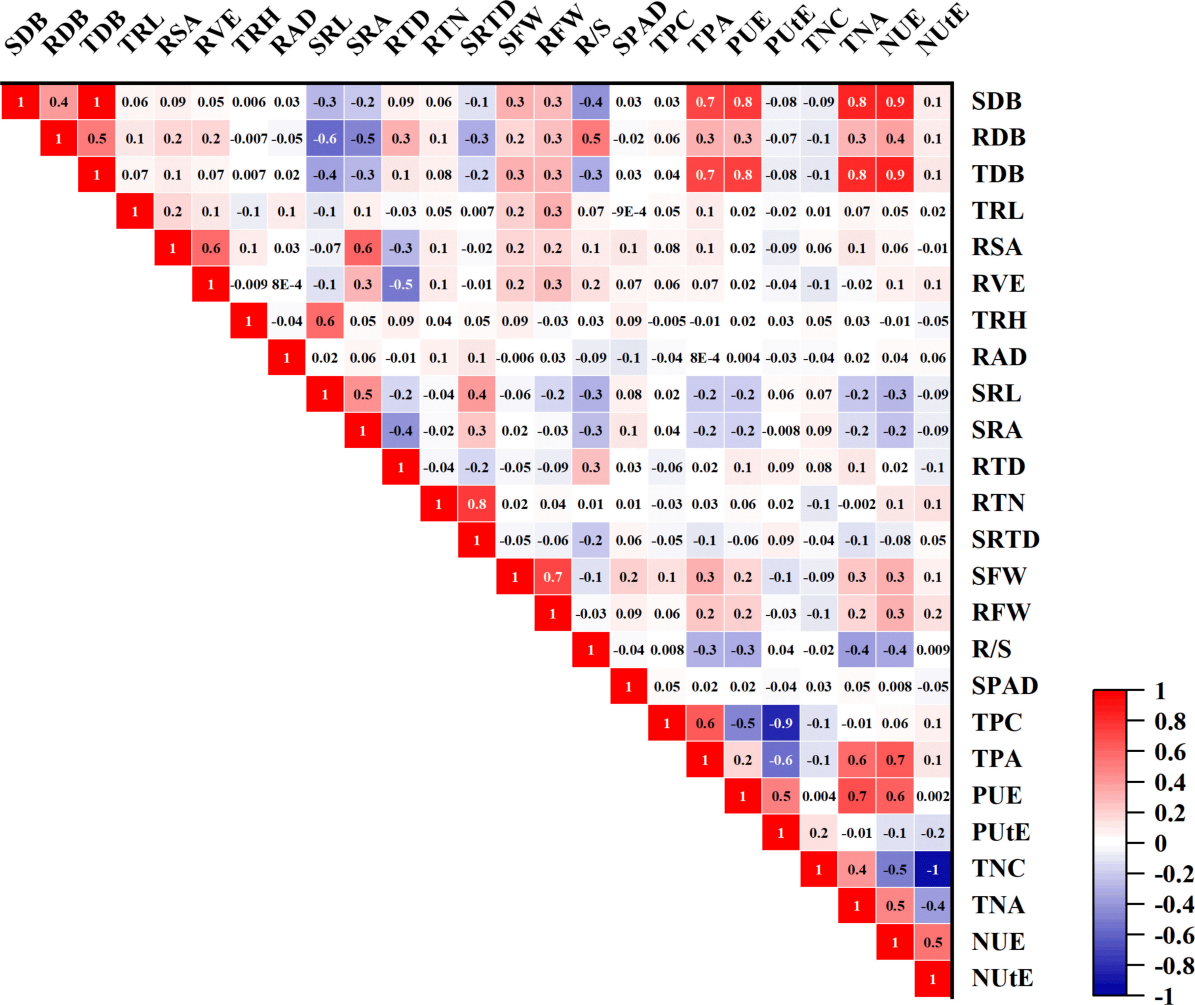
Pearson correlation study of 25 phenotypic traits in 384 Cotton Genotypes in relative values. The red box in the figure represents a positive correlation, the blue box represents a negative correlation, and the size of the number means the size of the correlation coefficient.

To find correlations between the traits, morphological traits’ relative values were correlated (Pearson test). There were significant connections for most of these traits (**Figure 7**). Among these traits, except for RTD and SRTD, there is a positive correlation between each root trait (**Figure 7**). There were significant negative correlations between RTD, RSA, and RVE, with correlation coefficients of -0.29 and -0.53, respectively, and a similar trend existed with SRL (**Figure 7**; **Supplementary Table S5**). In addition, except for SRA, SRTD, and SRL, root traits have positive correlations with SDB, but correlations between root traits and SDB are not obvious (**Figure 7**; **Supplementary Table S5**). The correlations of SDB with TDB, PUE, and NUE showed strong and highly positive correlation coefficients of 0.99, 0.77, and 0.87, respectively (**Figure 6**). A positive correlation was also observed between RDB with TDB, PUE, and NUE, with correlation coefficients of 0.52, 0.30, and 0.39, respectively. Additionally, a very low association between PUtE and morphological features suggests that this aspect of PUE needs to be addressed because the genotypes still have a tremendous potential for increased phosphorus use efficiency (**Figure 7**; **Supplementary Table S5**).

**Figure 8:**
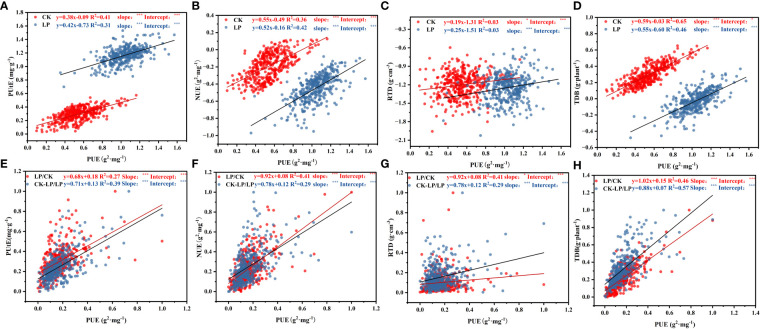
Standardized central axis linear relationships in morphological traits between PUE at both P supply and LP/CK, (CK-LP)/LP (Phosphorus stress index). Points are the mean values of four replicates per genotype. The data in figures **(A–D)** were log_10_ transformed before analysis, and the data in figures **(E–H)** were normalized before analysis. ∗∗∗P < 0.001. Trait notations: root tissue density (RTD), total dry biomass (TDB), nitrogen use efficiency (NUE), phosphorus use efficiency (PUE), phosphorus uptake efficiency (PUtE). * Indicates that the significance of the slope and intercept of the fitting curve of the two variables (p≤0.05).

Across cotton genotypes, most morphological traits related to root morphology and dry biomass traits covaried with PUE in response to LP conditions (**Figure 8**; **Supplementary Figure S3**). Significant and positive relationships between PUE and TDB were observed under both CK (R^2^ = 0.65, P<0.001) and LP (R^2^ = 0.46, P<0.05) conditions (**Figure 8**; **Supplementary Figures S2**, **S3**). However, there was a positive correlation between RDB and PUE under CK (R^2^ = 0.11, P<0.001) and LP (R^2^ = 0.20, P<0.0001) conditions, but it was not significant (**Figure 8**). The RSA and PUE were not significantly correlated under either CK (R^2^ = 0.01, P<0.001) and LP (R^2^ = 0.02, P<0.001) conditions (**Figure 8**). In addition, under the LP condition, there was a significant positive correlation between PUE with NUE (R^2^ = 0.65, P<0.001) and PUtE (R^2^ = 0.31, P<0.001), under the CK condition, have the same trends (**Figure 8**). The slopes in the relationships of PUtE, NUE, RTD, and TDB with PUE were significantly different between the two P supplies. Furthermore, the intercepts under CK compared to LP conditions were significantly higher (P<0.001) (**Figure 8**). Meanwhile, the RTD, TDB, PUtE, and NUE were positively correlated with PUE in the phosphorus stress index (LP/CK, CK-LP/LP) (**Figure 8**).

### Principle component analysis (PCA)

To find the response patterns of numerous traits and genotypes under various P supply conditions, the principal component analysis (PCA) was carried out. 25 features were obtained from the average of both cotton genotypes for the loading plots of PC1 and PC2 separately (**Supplementary Figures S4**, **S5**). In this study, seven principal components contributed to the total change in each trait and genotype at the two P supply levels (**Supplementary Tables S9**, **S10**). The cumulative variances of all PC interpolations under CK and LP were 81.68% and 79.25%, respectively (**Supplementary Tables S9**, **S10**). In CK conditions, the first two components, respectively, contributed 24.1% and 13.9% of the overall variation (**Supplementary Figure S4**). Under CK conditions, the SDB, TDB, PUE, NUE, and TNA scored high on the PC1; it can be considered the accumulation of biomass and phosphorus and nitrogen efficiency-related traits (**Supplementary Table S9**). The SRA, SRTD, RSA, and SRL scored high on the PC2 under CK conditions (**Supplementary Figure S4**, **Supplementary Table S9**). In LP conditions, PC1 represented 25.9% of the variability. It was dominated by biomass and phosphorus and nitrogen efficiency traits (e.g., SDB, RDB, TDB, TPA, PUE, TNA, and NUE) (**Supplementary Figure S5**, **Supplementary Table S10**). The PC2 represented 12.3% of the variance and primarily comprised TPC, TNC, and SRA (**Supplementary Figure S5**, **Supplementary Table S10**). Through principal component analysis, we found that the seven comprehensive indicators under two P supply conditions were mainly reflected in root-related traits and morphological indicators such as dry matter biomass (**Supplementary Tables S9**, **S10**). The result revealed that biomass and root traits governed maximum genetic variability (**Supplementary Figures S4**, **S5**; **Supplementary Tables S9**, **S10**). The cotton genotypes in quadrant I had higher biomass under both P supply conditions; contrarily, genotypes in quadrant II had higher PUE (**Supplementary Figures S4**, **S5**).

**Figure 9:**
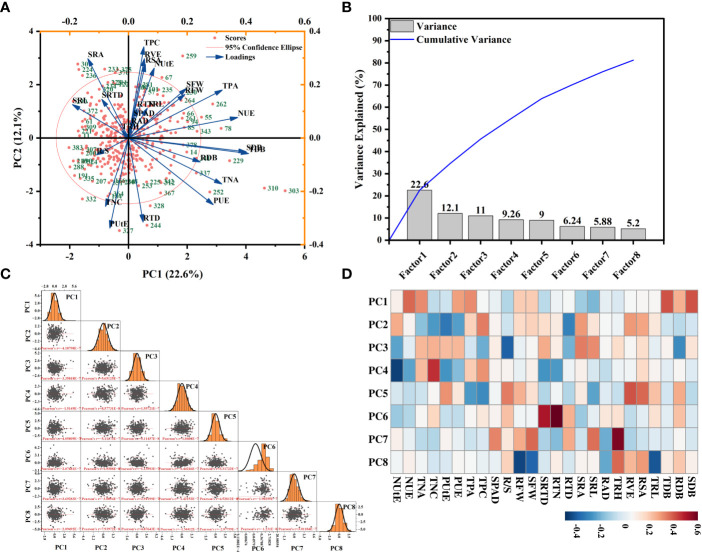
Principal component analysis (PCA) of morphological traits of 384 cotton genotypes in response to changes in each trait’s relative values. The first two principal components of scores **(A)** are indicated. The position of each genotype in the principal component space is indicated by a red circle, **(B)** a factor variance plot, **(C)** and score matrix of genotypes, and **(D)** principle factors heatmap. Genotype names are replaced with numbers, and the genotypes represented by each number correspond to those described in **Supplementary Table 1**. Points are the mean values with replicates per genotype.

In this study, eight principal component factors were extracted from PCA analysis based on the relative values of each morphological trait (**Supplementary Table S8**). The first two components contributed 22.6% and 12.1% of the overall variation (**Figure 9**). The morphological traits that explained genotypic variation in PC1 were TDB, SDB, TPA, PUE, TNA, and NUE, which can be considered as the accumulation of biomass and phosphorus and nitrogen efficiency-related traits (**Figure 9**, **Supplementary Table S8**). Within PC2, the maximum genotypic variation was contributed by RSA, RVE, and SRA, and it can be considered the root morphological traits (**Figure 9**, **Supplementary Table S8**).

## Discussion

### P availability impact on plant growth index and phosphorus and nitrogen efficiency index

In this research, there were significant differences in dry matter accumulation of cotton under LP and CK conditions. The TDB decreased by 51.95% in LP conditions compared to CK conditions (**Figure 1B**, **Supplementary Table S2**). These results are consistent with previous studies, which found that low phosphorus stress can significantly reduce the total dry weight of wheat (de Souza Campos et al., 2019). According to research on wheat, P absorption reduces when the total dry weight falls (Soumya et al., 2020). The SDB and TPA of whole plants were dramatically lowered under LP conditions in our study (**Figures 1A**, **3C**). These findings concur with some of those reported by (Duan et al., 2020). Low P stress frequently changed the root system by enhancing root morphological features and increasing biomass allocation to roots (Lugli et al., 2021). In this study, the RDB is higher under LP conditions than under CK conditions (**Supplementary Table S2**). Previous studies concluded that root cells were under more respiration stress due to the increased biomass partitioning to roots at low P concentrations (Prince et al., 2019; Chen et al., 2020). Moreover, LP conditions significantly enhanced the R/S ratio in this study (**Supplementary Table S2**). Previous research found that the increased allocation of photosynthates to roots, which leads to an increased root-to-shoot ratio and P acquisition, was linked to how crop plants responded to P deprivation (Han et al., 2021). In plants with a P shortage, it was found that allocating biomass to root development improves P uptake (Brown and Linch, 2001; Wen et al., 2019). Due to a decline in shoot growth and an increase in root growth in wheat, an elevated R/S ratio was noted under the LP conditions (Shen et al., 2018).

In this study, the decreasing PUE under CK conditions may result from the accumulation of external phosphorus beyond what is required for growth, negatively impacting a plant’s P economy (**Supplementary Table S2**). This indicates that while plants can absorb more P than they need, external phosphorus cannot constantly encourage growth (Julia et al., 2018; Adem et al., 2020). In this study, no changes in NUE were seen in cotton genotypes when plants were grown under both nutrition circumstances (**Figure 3F**, **Supplementary Table S2**). Our results are consistent with a prior study on plants growing in acidic, high-P-fixing soil in this regard (Seguel et al., 2017). In LP circumstances, cotton genotypes exhibit a more significant phosphorus and nitrogen uptake efficiency (**Figures 3G, H**). The previous study finds that the common plant adaptations to P deficiency involve changes to root growth to increase P uptake (Haling et al., 2016; Hayes et al., 2018).

### Effects of P supply on cotton root morphological traits

**Figure 10:**
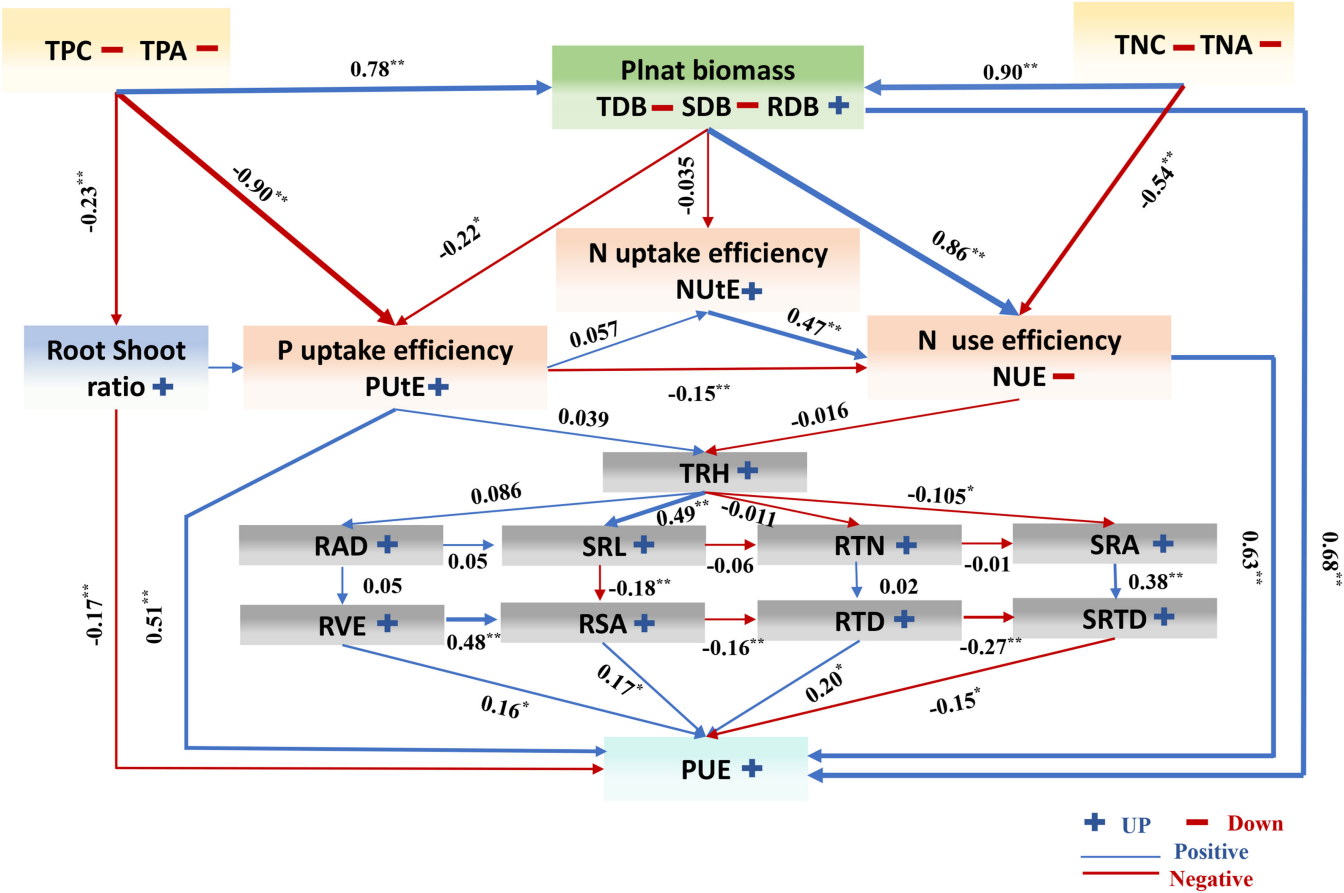
A working model of the response of cotton to low phosphorus availability. The colors of the arrows indicate that the morphological indicators were up (blue) or down (red), and the correlation line is based on Pearson coefficients. Trait notations: shoot dry biomass (SDB), root dry biomass (RDB), total dry biomass (TDB), taproot length (TRL), root surface area(RSA), root volume (RVE), total root length (TRH), root average diameter (RAD), specific root length (SRL), specific root area (SRA), root tissue density(RTD), root tips number (RTN), specific root tips density (SRTD), shoot fresh weight (SFW), root fresh weight (RFW), root shoot ratio (R/S), SPAD value (SPAD), total phosphorus concentration (TPC), total phosphorus accumulation (TPA), phosphorus use efficiency (PUE), phosphorus uptake efficiency (PUtE), total nitrogen concentration (TNC), total nitrogen accumulation (TNA), nitrogen use efficiency (NUE), nitrogen uptake efficiency (NUtE). * indicates the significance of the correlation coefficient between different variables, the * sign indicates that the significance of the correlation coefficient between the two variables is less than 0.05, and ** indicates that the p value is less than 0.01. The blue and red lines respectively represent the negative and positive relationship between different variables. The number represents the path coefficient between different variables; and * and ** represent P < 0.05 and P < 0.01, respectively.

Most plants will alter their behavior in response to various nutrient levels (Abdolzadeh et al., 2010). Roots are essential for adjusting to variations in P availability and can change the plant’s root system (Peret et al., 2011; Zhang et al., 2014). In this study, under various phosphorus availability, root properties had a high genotypic variance (**Figure 2**). Our study showed that cotton genotypes’ root morphological characteristics were significantly increased under low P conditions (**Figure 10**). In previous research, in response to varying levels of P supply, rice and chickpea *(Cicer arietinum* L.) germplasm accessions exhibit a surprising degree of variety in root shape (Mori et al., 2016; Chen et al., 2017). Based on our study, the genotypes of RAD in CK circumstances varied between 0.36 and 1.44 mm, Whereas, under LP circumstances, the cotton genotypes ranged from 0.37 to 1.07 mm (**Supplementary Table S2**). Studies on Arabidopsis and rice demonstrate that increasing the RTD under low P stress can significantly increase the capacity of P to be absorbed (Williamson, 2001; Fitter et al., 2002). In this study, the RTD of cotton genotypes under LP circumstances increased by 7.88% compared to CK conditions (**Supplementary Table S2**). As a result, plant cultivars with high RTD are may more suitable for LP conditions.

In this study, LP conditions highly impact the SRL (**Figure 2C**). According to other research reports, bamboo increases P by expanding its foraging range and SRL (Yang et al., 2020). Similarly, low P stress increases root growth in pepper plants, increasing SRL (Wen et al., 2020). In this research, LP conditions markedly raised the cotton genotypes TRH. Compared to CK conditions, the LP conditions increased by 5.69%, respectively (**Figure 2B**), consistent with earlier results (Lynch, 2011). Wheat also improved TRH under LP circumstances (Shen et al., 2018; Lal et al., 2019). The RSA significantly impacts by genotypic diversity at various P supply levels, it may contribute to the observed rise in TRH and RSA, which enhances P availability (Liu et al., 2015). Previous studies discovered that root tips are a key factor in improving P uptake in Arabidopsis (Fitter et al., 2002). Our analysis also finds that, in LP conditions, RTN will increase significantly (**Supplementary Table S2**). The previous study found that under P deprivation, root biomass switched in P-efficient common bean genotypes to increase the RTN (Gahoonia and Nielsen, 2004). Previous studies suggest that improved TRH and RTN are the adaption change that improves P acquisition in LP conditions (Gutierrez-Alanis et al., 2018).

### The correlation of morphological traits and effect on phosphorus use efficiency

The PCA result revealed the most significant trait contributing to overall variation (**Figure 9**). Our findings align with earlier research showing that P stress changes wheat TRL, SDB, RSA, and RTN (Bilal et al., 2018), as well as the RSA and TRL in common beans (Rangarajan et al., 2018). Under both P supply levels, we find that the SDB was positively connected with the RDB and TDB. In previous studies of wheat under LP stress, the RDB and SDB also positively correlated (Chen et al., 2020). In our research, under LP stress, the TDB and RDB were substantially linked with root characteristics; this is consistent with previous studies in rice (Vejchasarn et al., 2016). We also find that SDB was positively correlated with RTD and negatively correlated with SRL under the LP condition (**Supplementary Figure S3**). This indicates that under LP conditions, root traits play different roles in dry matter accumulation above ground, and the impact of root traits on cotton resource acquisition in a low P environment needs further exploration.

The root traits have a significant positive correlation with RDB in our study. It may be that plants can improve the uptake and use of phosphorus by changing the root morphological traits to increase the RDB. Our results showed that RTD, NUE, PUtE, and TDB were significantly and positively correlated with PUE (**Figures 7**, **8**). Rice led the same result under various P conditions; PUE was also positively linked with TDB (Wissuwa et al., 2020). Our study showed that genotypes with higher TDB and RDB performed more effectively in improving PUtE, while in other research, the RTN, RSA, TRL, and TRH were significant in PUtE (Sanguineti et al., 2007; Honvault et al., 2021). In rice, the RDB and RSA under LP conditions were the cause of the genotypic variance in PUE (Mori et al., 2016). We did find a significant association between PUtE and PUE under various P supply conditions, suggesting that these traits are interrelated rather than separately inherited at the seedling stage (**Figures 7**, **8**). Although there is no significant correlation between TPC and PUE among cotton varieties, we found that there is a positive correlation between TPC and PUtE (**Figure 7**), which may indicate that the phosphorus content in cotton will directly affect the phosphorus absorption of cotton, which was also found in previous studies (Chu et al., 2020).

### Plasticity of multiple morphological traits response to P availability

Recently, increased focus on how plants respond to complex environments has led to a more precise knowledge of the genetic variability that still exists in plants that is beneficial for selection and serves as a supplement to genetic heredity (Lacey et al., 2021; Wang et al., 2021). In response to various nutritional changes, plant morphological features exhibit significant plasticity, and P availability specifically influences root shape. However, modifications to root shape are thought to be a prevalent tactic to improve P acquisition (Duan et al., 2020). In this study, the cotton genotypes with well-developed root systems would exhibit more remarkable plasticity in response to LP conditions, especially in improving the morphological characteristics of the root system (**Figure 6**). Previous studies found that thick-rooted species (like chickpeas) showed restricted root morphological plasticity in response to the availability of soil resources (P) (Li et al., 2014; Eissenstat et al., 2015; Lyu et al., 2016; Wen et al., 2019).

It was frequently the case that high disparities indicated traits with low heritability and great adaptability in h^2^ calculated in various situations. These conflicting characteristics between plasticity and heritability have already been examined (Kusmec et al., 2017). In this study, except for the enormous genetic variety taken into account, the most outstanding PL values were the biomass traits (TDB, SDB, SFW, RFW), TRH, SRL, and TPC (**Figure 6**). In contrast, RVE and RSA displayed h^2^B values >0.7 under both P levels and the lowest PL values for root morphological traits (**Figure 6**). The fact that traits with weaker plasticity usually exhibited high plastic heritability, which was caused by a sizeable genetic variance, highlighted a lingering genetic variability relevant for selection (Lacey et al., 2021). Furthermore, it is interesting that PUE and PUtE seemed substantially linked with RDB, RTD, and RSA, all of which had h^2^B values > 0.7 under both nutrition levels (**Figure 6**). These results supported the hypothesis that root system architecture (RSA) influences a plant’s ability to uptake P and may impact PUE (Lyu et al., 2016).

## Conclusion

A simplified working model of the response of cotton to LP conditions is provided in (**Figure 10**). The morphological traits of 384 cotton genotypes studied in this study showed significant genotype differences in response to changes in P availability. These changes increased root traits and maximized PUE. In this study, LP conditions inhibited the accumulation of phosphorus and dry matter in cotton seedlings. We found that root morphological traits greatly influenced cotton biomass traits, PUE. The results show that TDB, RTD, NUE, and PUtE are the essential indexes affecting and increasing PUE under LP conditions. Cotton genotypes show large plasticity (PL) in root morphological traits, which is one of the critical responses of cotton to adapt to LP conditions. Finally, this study shows that optimizing root phenotype is an important way for plants to increase PUE under LP conditions. Our results provide suggestions for future research to enhance the ability of the earth system model to predict how crops respond to environmental interference and provide target quality for cotton breeding in phosphorus-deficient areas.

## Data availability statement

The original contributions presented in the study are included in the article/**Supplementary Materials**. Further inquiries can be directed to the corresponding authors.

## Author contributions

MK: Writing – original draft, Conceptualization, performed the experiments. XL: Formal analysis Data. AI: Formal analysis Data. XW: Analysis Data. HG: Investigation. QQ: Investigation. SR: Investigation. RG Investigation. QD: conceived and designed the experiments. XZ: Resources, Supervision, review and editing. MS: Resources, Supervision, review and editing. All authors read and approved the final manuscript.

## Funding

We greatly appreciate financial support from the State Key Laboratory of Cotton Biology, Institute of Cotton Research of CAAS (CB2021C10) and the Central Public-interest Scientific Institution Basal Research Fund, CAAS (Y2021XK12).

## Conflict of interest

The authors declare that the research was conducted in the absence of any commercial or financial relationships that could be construed as a potential conflict of interest.

## Publisher’s note

All claims expressed in this article are solely those of the authors and do not necessarily represent those of their affiliated organizations, or those of the publisher, the editors and the reviewers. Any product that may be evaluated in this article, or claim that may be made by its manufacturer, is not guaranteed or endorsed by the publisher.
