# Supplementary material for: Genetic variation in morphological traits in cotton and their roles in increasing phosphorus-use-efficiency in response to low phosphorus availability
Source: Front Plant Sci. 2022 Nov 30;13:1051080. doi: 10.3389/fpls.2022.1051080 (PMC9749730; doi:10.3389/fpls.2022.1051080)
Supplement: Supplementary file 1 [file DataSheet_1.docx]

Supplementary Material

# Supplementary Figures and Tables

## Supplementary Figures

**
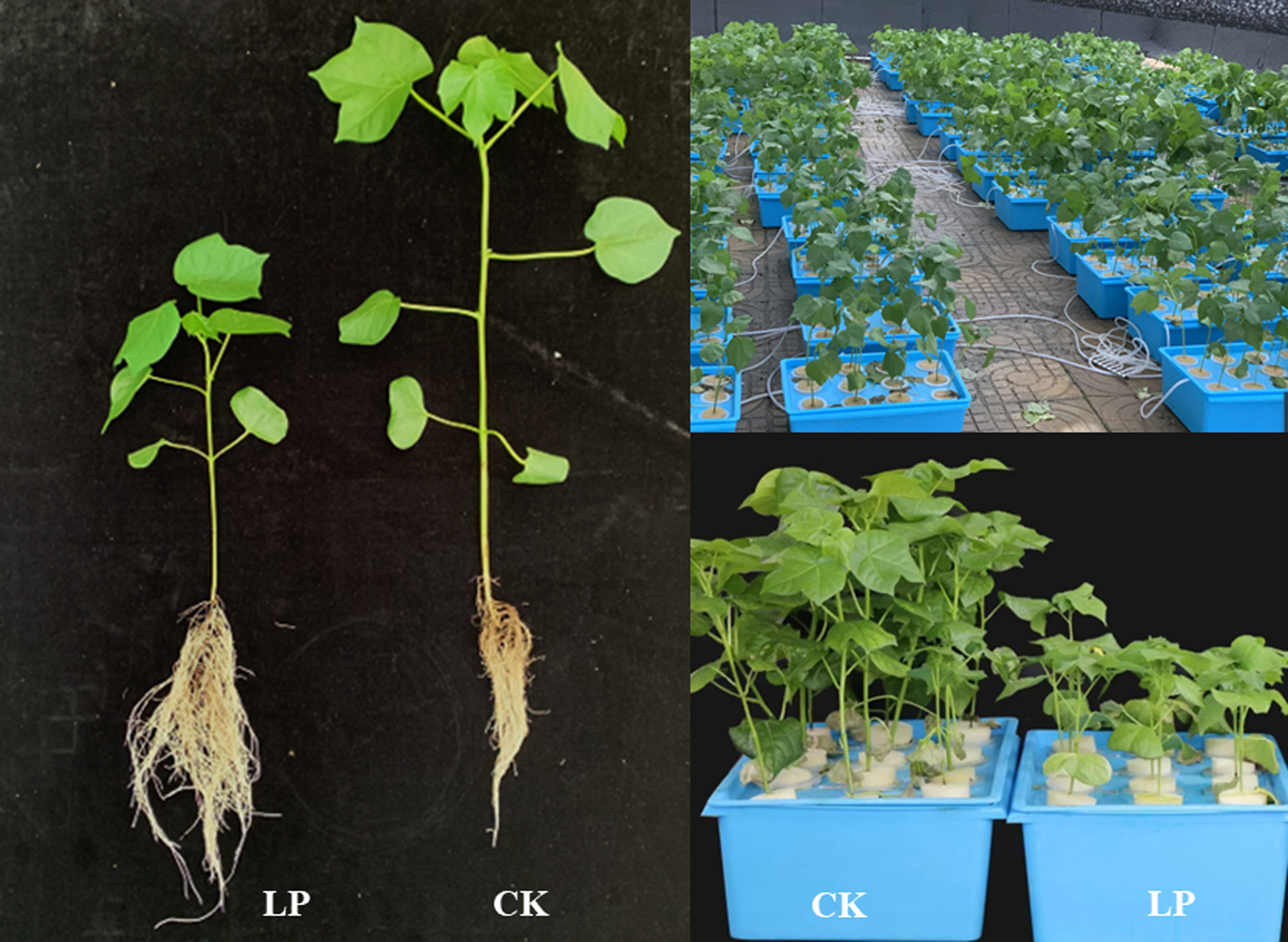
**

**Supplementary Figure 1.** Experimental layout and a close-up view of cotton plants grown in the hydroponic phenotyping platform for characterizing trait variability in the germplasm glasshouse, with an example of two contrasting genotypes grown for 28 d.


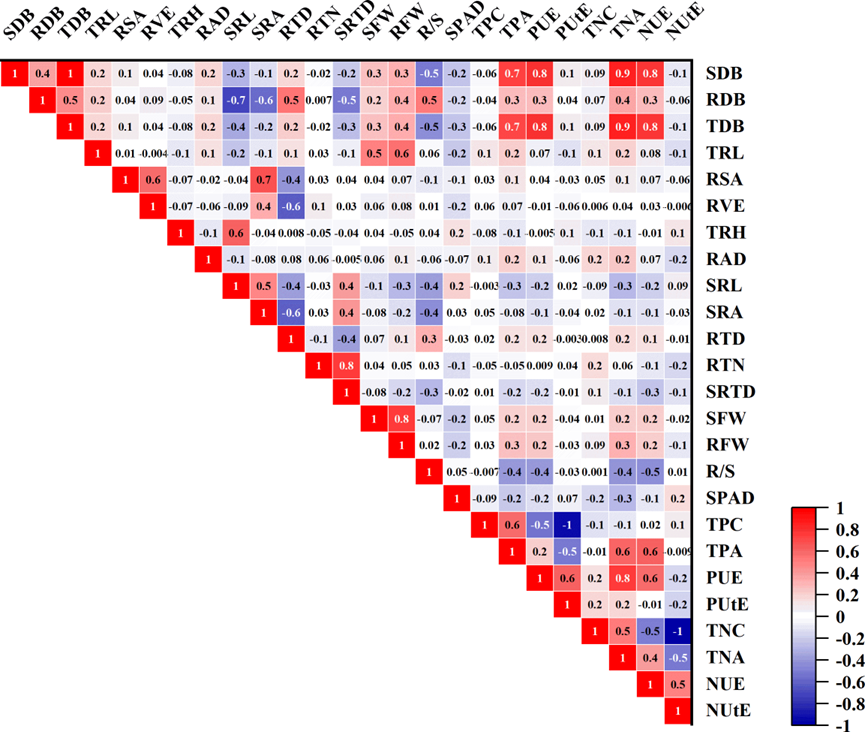


**Supplementary Figure 2.** Pearson correlation study of 25 phenotypic traits in 384 Cotton Genotypes under CK levels. The red box in the figure represents positive correlation, the blue box represents negative correlation, and the size of the number represents the size of the correlation coefficient.

**

**

**Supplementary Figure 3.** Pearson correlation study of 25 phenotypic traits in 384 Cotton Genotypes under LP levels. The red box in the figure represents positive correlation, the blue box represents negative correlation, and the size of the number represents the size of the correlation coefficient.


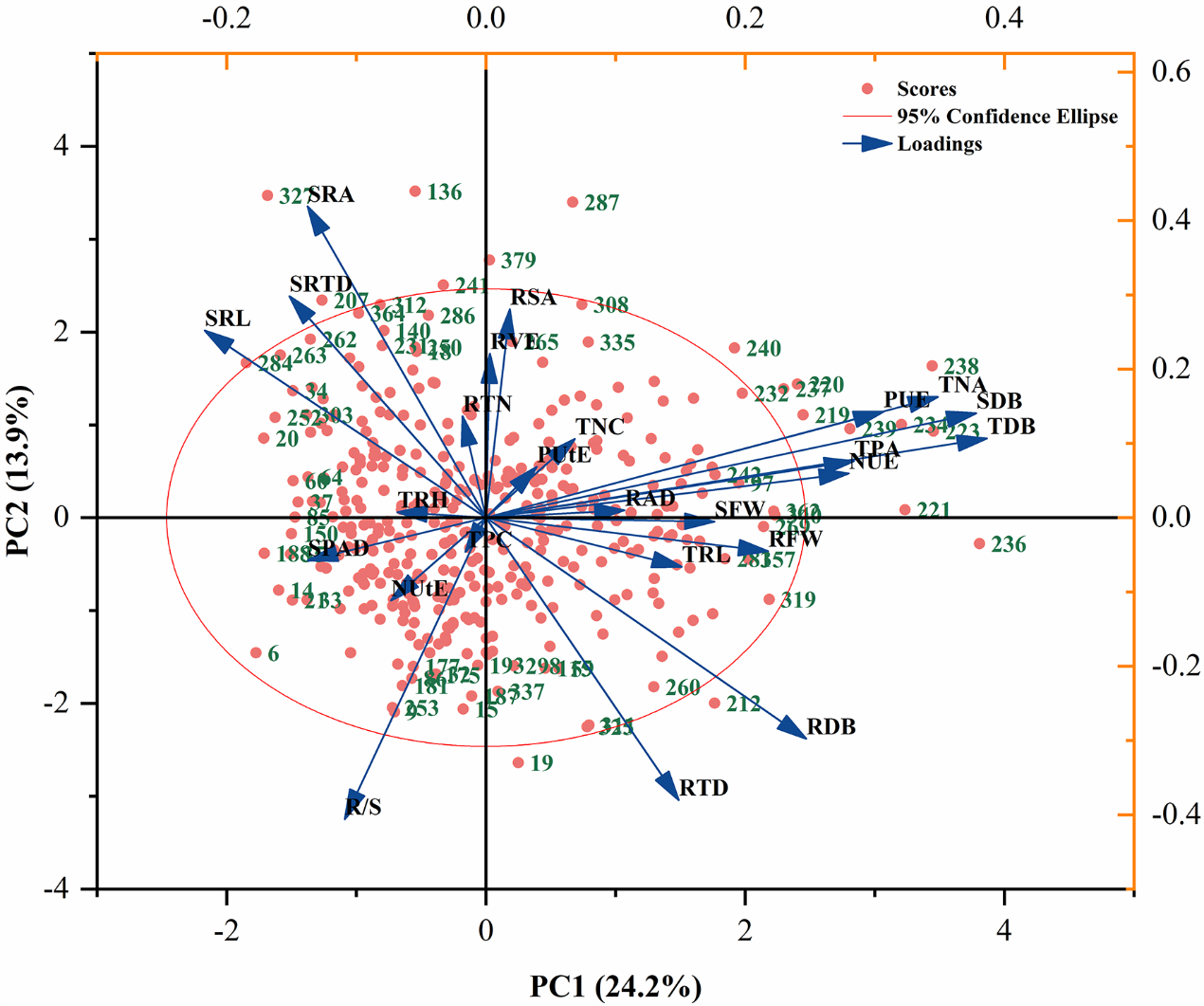


**Supplementary Figure 4.** Principal component analysis (PCA) of morphological traits of 384 cotton genotypes in response to changes in CK conditions. The position of each genotype in the principal component space is indicated by a red circle. Genotype names are replaced with numbers, and the genotypes represented by each number correspond to those described in supplementary table S1. Points are the mean values with replicates per genotype.


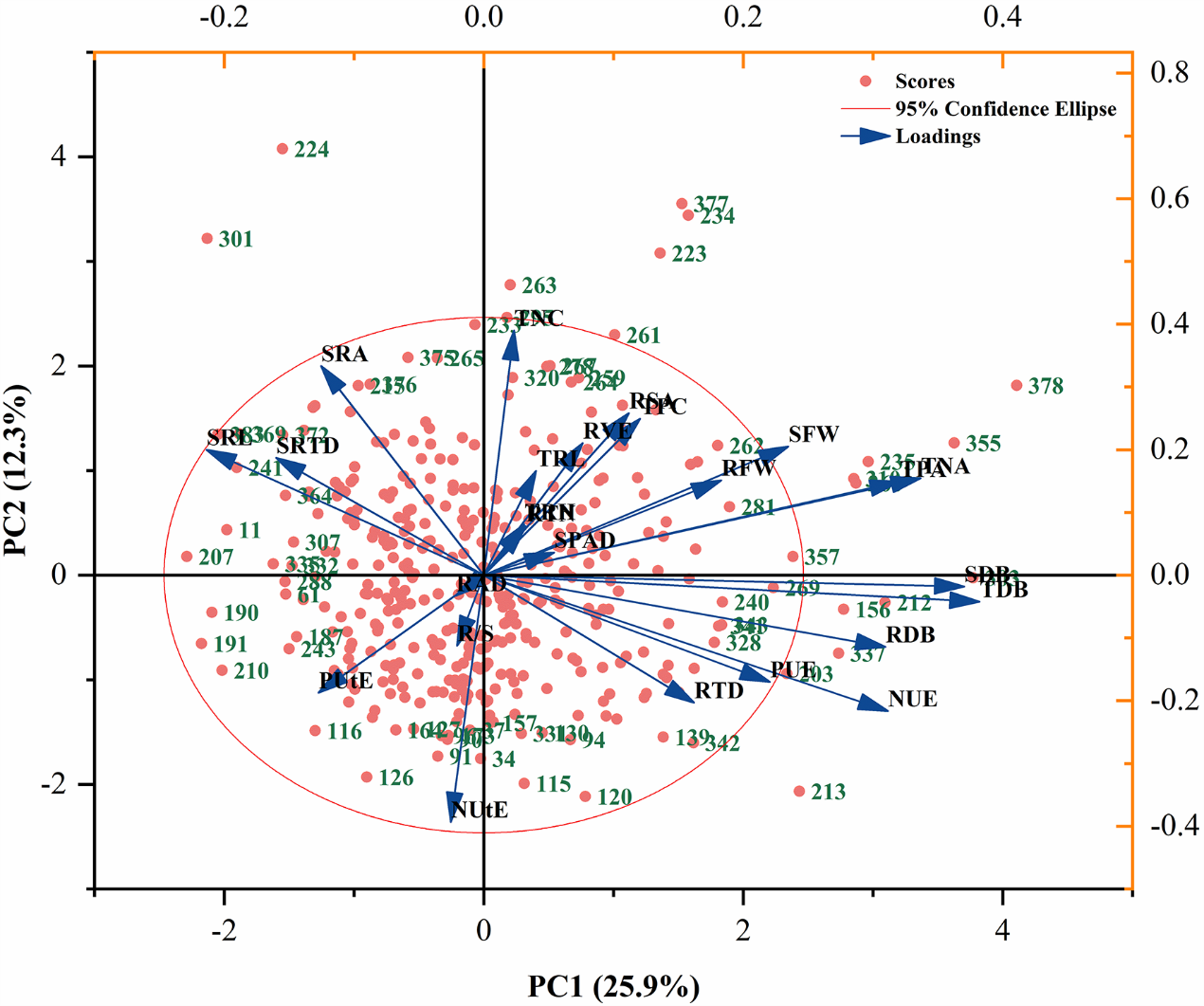


**Supplementary Figure 5.** Principal component analysis (PCA) of morphological traits of 384 cotton genotypes in response to changes in LP conditions. The position of each genotype in the principal component space is indicated by a red circle. Genotype names are replaced with numbers, and the genotypes represented by each number correspond to those described in supplementary table S1. Points are the mean values with replicates per genotype.

## Supplementary Tables

**Supplementary Table 4** Heritability (broad sense, h^2^B of each trait calculated in CK and LP conditions. plasticity (PL) and plastic heritability(h^2^PL) were also estimated taking into account G x N interaction

| **Index** | **CK_H^2^B** | **LP_H^2^B** | **H^2^B at LP/CK** | **Plasticity（PL)** | **Plastic heritability H^2^(PL)** |
| --- | --- | --- | --- | --- | --- |
| Shoot dry biomass  Root dry biomass  Total dry biomass  Taproot length | 0.9965  0.9960  0.9960  0.9800 | 0.9964  1.0000  0.9970  0.9900 | 0.9999  1.0040  1.0010  1.0102 | 0.9989  0.4670  0.9990  0.3740 | 0.0008  0.3930  0.0010  0.1790 |
| Root surface area  Root volume  Root total length  Root average diameter  Specific root length  Specific root area  Root tissue density  Root tips number  Specific root tips density  Shoot fresh weight | 0.9970  0.9983  0.9950  1.0000  0.9978  0.7930  0.6893  0.9980  0.9981  0.9900 | 0.9979  0.9980  0.9959  1.0000  0.9982  0.7311  0.6914  0.9985  0.9993  0.9900 | 1.0009  0.9998  1.0009  1.0000  1.0004  0.9220  1.0031  1.0005  1.0007  1.0000 | 0.4567  0.4633  0.9562  0.9569  0.8836  0.8509  0.8606  0.9846  0.9771  0.9900 | 0.2567  0.2551  0.0365  0.0422  0.1171  0.1343  0.1309  0.0174  0.0191  0.1530 |
| Root fresh weight  Root shoot ratio  SPAD Value | 0.9970  0.9964  0.9100 | 0.9960  0.9954  0.9900 | 0.9990  0.9990  1.0879 | 0.9709  0.9991  0.3300 | 0.0090  0.0008  0.1790 |
| Total P concentration  Total P accumulation  P use efficiency | 0.9992  0.9999  0.9941 | 0.9997  0.9999  0.9723 | 1.0005  1.0001  0.9782 | 0.9998  0.9995  0.9997 | 0.0002  0.0004  0.0003 |
| P uptake efficiency  Total N concentration | 0.9828  0.9944 | 0.9830  0.9923 | 1.0003  0.9978 | 0.9985  0.9969 | 0.0012  0.0018 |
| Total N accumulation  N use efficiency  N uptake efficiency | 0.9974  0.9954  0.9983 | 0.9973  0.9927  0.9983 | 0.9999  0.9972  0.9999 | 0.9988  0.9963  0.9988 | 0.0009  0.0021  0.0009 |

| **Supplementary Table 8** Principal component analysis of 25 selected traits and the proportion of variation in each principal component under LP/CK | | | | | | | | |
| --- | --- | --- | --- | --- | --- | --- | --- | --- |
| **Principle factors** | **LP/CK** | | | | | | | |
|  | **PC1** | **PC2** | **PC3** | **PC4** | **PC5** | **PC6** | **PC7** | **PC8** |
| **SDB** | 0.40 | -0.05 | 0.13 | -0.01 | -0.09 | -0.03 | -0.04 | 0.08 |
| **RDB** | 0.24 | -0.09 | -0.30 | 0.06 | 0.27 | 0.22 | 0.06 | 0.21 |
| **TDB** | 0.41 | -0.06 | 0.07 | -0.01 | -0.05 | 0.00 | -0.03 | 0.10 |
| **TRL** | 0.06 | 0.11 | 0.00 | 0.07 | 0.22 | 0.14 | -0.02 | -0.42 |
| **RSA** | 0.06 | 0.28 | 0.15 | 0.17 | 0.37 | 0.00 | -0.06 | 0.30 |
| **RVE** | 0.05 | 0.30 | 0.06 | 0.04 | 0.40 | -0.09 | -0.12 | 0.24 |
| **TRH** | -0.02 | 0.03 | 0.15 | 0.02 | -0.08 | 0.14 | 0.57 | 0.37 |
| **RAD** | 0.01 | 0.05 | 0.06 | -0.09 | -0.02 | 0.11 | -0.21 | -0.22 |
| **SRL** | -0.19 | 0.13 | 0.32 | -0.04 | -0.24 | 0.00 | 0.36 | 0.08 |
| **SRA** | -0.14 | 0.30 | 0.34 | 0.08 | 0.11 | -0.15 | -0.07 | 0.07 |
| **RTD** | 0.05 | -0.32 | -0.20 | 0.03 | -0.11 | 0.25 | 0.26 | -0.03 |
| **RTN** | 0.03 | 0.11 | 0.10 | -0.27 | 0.03 | 0.63 | -0.18 | 0.09 |
| **SRTD** | -0.09 | 0.15 | 0.26 | -0.27 | -0.10 | 0.50 | -0.12 | -0.04 |
| **SFW** | 0.20 | 0.19 | 0.06 | 0.08 | 0.13 | 0.04 | 0.37 | -0.38 |
| **RFW** | 0.19 | 0.16 | 0.02 | 0.02 | 0.25 | 0.04 | 0.26 | -0.45 |
| **R/S** | -0.11 | -0.06 | -0.39 | 0.07 | 0.34 | 0.22 | 0.12 | 0.19 |
| **SPAD** | 0.02 | 0.08 | 0.10 | 0.08 | 0.04 | 0.07 | 0.33 | 0.02 |
| **TPC** | 0.05 | 0.34 | -0.21 | 0.30 | -0.29 | 0.11 | -0.03 | 0.02 |
| **TPA** | 0.32 | 0.18 | -0.05 | 0.20 | -0.26 | 0.06 | -0.06 | 0.05 |
| **PUE** | 0.29 | -0.25 | 0.24 | -0.19 | 0.12 | -0.09 | 0.01 | 0.06 |
| **PUtE** | -0.06 | -0.34 | 0.23 | -0.28 | 0.30 | -0.07 | 0.08 | -0.03 |
| **TNC** | -0.08 | -0.26 | 0.25 | 0.46 | 0.06 | 0.17 | -0.10 | -0.05 |
| **TNA** | 0.32 | -0.17 | 0.24 | 0.23 | -0.06 | 0.06 | -0.09 | 0.04 |
| **NUE** | 0.37 | 0.08 | -0.01 | -0.24 | -0.09 | -0.10 | 0.01 | 0.08 |
| **NUtE** | 0.09 | 0.26 | -0.24 | -0.47 | -0.04 | -0.15 | 0.08 | 0.04 |
| **Eigenvalue** | 5.65 | 3.02 | 2.75 | 2.31 | 2.25 | 1.56 | 1.47 | 1.30 |
| **Percentage of Variance (%)** | 22.59 | 12.07 | 11.01 | 9.26 | 9.00 | 6.24 | 5.88 | 5.20 |
| **Cumulative (%)** | 22.59 | 34.65 | 45.66 | 54.92 | 63.92 | 70.16 | 76.05 | 81.25 |

25 morphological traits with CV (**Supplementary Table 2**) were used for factor analysis using the principal component analysis (PCA) extraction method. For each trait, the largest variable loading score crossing the two components appears in bold. Principal components with eigenvalues > 1 are presented and considered significant.

**Supplementary Table 9** Principal component analysis of 25 selected traits and the proportion of variation in each principal component under CK condition

|  | | | | | | | |
| --- | --- | --- | --- | --- | --- | --- | --- |
| **Principle factors** | **CK** | | | | | | |
|  | **PC1** | **PC2** | **PC3** | **PC4** | **PC5** | **PC6** | **PC7** |
| **SDB** | 0.38 | 0.14 | 0.04 | -0.12 | -0.07 | -0.05 | 0.02 |
| **RDB** | 0.25 | -0.30 | -0.05 | 0.08 | 0.22 | -0.13 | 0.08 |
| **TDB** | 0.39 | 0.11 | 0.04 | -0.11 | -0.04 | -0.06 | 0.03 |
| **TRL** | 0.15 | -0.07 | 0.03 | 0.27 | 0.07 | 0.37 | -0.11 |
| **RSA** | 0.02 | 0.28 | 0.09 | 0.15 | 0.40 | -0.20 | -0.10 |
| **RVE** | 0.00 | 0.22 | 0.09 | 0.18 | 0.46 | -0.17 | 0.02 |
| **TRH** | -0.07 | 0.01 | 0.00 | -0.21 | -0.06 | 0.27 | -0.33 |
| **RAD** | 0.11 | 0.01 | -0.03 | 0.13 | -0.17 | -0.05 | 0.03 |
| **SRL** | -0.22 | 0.25 | 0.04 | -0.18 | -0.21 | 0.25 | -0.27 |
| **SRA** | -0.14 | 0.42 | 0.09 | 0.06 | 0.13 | -0.04 | -0.13 |
| **RTD** | 0.15 | -0.38 | -0.07 | -0.06 | -0.23 | 0.03 | 0.03 |
| **RTN** | -0.02 | 0.14 | -0.13 | 0.19 | -0.07 | 0.20 | 0.62 |
| **SRTD** | -0.15 | 0.30 | -0.07 | 0.12 | -0.20 | 0.22 | 0.45 |
| **SFW** | 0.18 | -0.01 | 0.06 | 0.16 | 0.16 | 0.53 | -0.12 |
| **RFW** | 0.22 | -0.04 | 0.02 | 0.20 | 0.19 | 0.43 | -0.11 |
| **R/S** | -0.11 | -0.41 | -0.11 | 0.17 | 0.24 | -0.06 | 0.05 |
| **SPAD** | -0.14 | -0.06 | 0.01 | -0.21 | -0.04 | -0.05 | -0.13 |
| **TPC** | -0.02 | -0.05 | 0.42 | 0.31 | -0.25 | -0.09 | -0.07 |
| **TPA** | 0.28 | 0.08 | 0.31 | 0.11 | -0.21 | -0.12 | -0.03 |
| **PUE** | 0.31 | 0.14 | -0.21 | -0.26 | 0.06 | 0.02 | 0.04 |
| **PUtE** | 0.04 | 0.07 | -0.44 | -0.31 | 0.21 | 0.08 | 0.04 |
| **TNC** | 0.07 | 0.11 | -0.40 | 0.31 | -0.18 | -0.13 | -0.22 |
| **TNA** | 0.35 | 0.16 | -0.15 | 0.04 | -0.14 | -0.11 | -0.09 |
| **NUE** | 0.28 | 0.06 | 0.28 | -0.29 | 0.05 | 0.03 | 0.16 |
| **NUtE** | -0.07 | -0.11 | 0.39 | -0.32 | 0.19 | 0.12 | 0.22 |
| **Eigenvalue** | 6.04 | 3.47 | 3.03 | 2.70 | 1.99 | 1.73 | 1.47 |
| **Percentage of Variance (%)** | 24.15 | 13.88 | 12.11 | 10.80 | 7.97 | 6.91 | 5.87 |
| **Cumulative (%)** | 24.15 | 38.03 | 50.14 | 60.93 | 68.90 | 75.81 | 81.68 |

25 morphological traits with CV (**Supplementary Table 2**) were used for factor analysis using the principal component analysis (PCA) extraction method. For each trait, the largest variable loading score crossing the two components appears in bold. Principal components with eigenvalues > 1 are presented and considered significant.

| **Supplementary Table 10** Principal component analysis of 25 selected traits and the proportion of variation in each principal component under LP condition | | | | | | | |
| --- | --- | --- | --- | --- | --- | --- | --- |
| **Principle factors** | **LP** | | | | | | |
|  | **PC1** | **PC2** | **PC3** | **PC4** | **PC5** | **PC6** | **PC7** |
| **SDB** | 0.37 | -0.02 | 0.02 | -0.02 | 0.20 | -0.02 | 0.00 |
| **RDB** | 0.31 | -0.11 | 0.07 | 0.20 | -0.23 | 0.15 | 0.08 |
| **TDB** | 0.38 | -0.04 | 0.03 | 0.03 | 0.11 | 0.02 | 0.02 |
| **TRL** | 0.04 | 0.17 | 0.23 | 0.05 | -0.18 | 0.11 | 0.11 |
| **RSA** | 0.11 | 0.26 | 0.31 | -0.02 | -0.16 | -0.06 | 0.32 |
| **RVE** | 0.08 | 0.21 | 0.34 | -0.10 | -0.21 | -0.10 | -0.11 |
| **TRH** | 0.03 | 0.08 | -0.02 | 0.24 | 0.18 | -0.03 | 0.47 |
| **RAD** | -0.02 | -0.03 | 0.18 | -0.10 | 0.03 | 0.19 | 0.45 |
| **SRL** | -0.21 | 0.20 | -0.06 | -0.01 | 0.37 | -0.02 | 0.24 |
| **SRA** | -0.13 | 0.33 | 0.24 | -0.17 | 0.08 | -0.09 | 0.20 |
| **RTD** | 0.16 | -0.20 | -0.24 | 0.28 | 0.02 | 0.18 | 0.16 |
| **RTN** | 0.03 | 0.08 | 0.15 | 0.03 | 0.07 | 0.71 | -0.17 |
| **SRTD** | -0.16 | 0.19 | 0.09 | -0.09 | 0.29 | 0.52 | -0.14 |
| **SFW** | 0.23 | 0.21 | 0.04 | -0.18 | -0.01 | -0.07 | -0.10 |
| **RFW** | 0.18 | 0.15 | 0.21 | -0.16 | -0.22 | -0.03 | -0.10 |
| **R/S** | -0.02 | -0.11 | 0.07 | 0.29 | -0.52 | 0.19 | 0.08 |
| **SPAD** | 0.05 | 0.04 | 0.02 | -0.22 | -0.02 | -0.02 | -0.37 |
| **TPC** | 0.12 | 0.25 | -0.40 | -0.21 | -0.16 | 0.11 | 0.13 |
| **TPA** | 0.32 | 0.15 | -0.23 | -0.13 | 0.04 | 0.06 | 0.08 |
| **PUE** | 0.22 | -0.17 | 0.32 | 0.16 | 0.30 | -0.10 | -0.08 |
| **PUtE** | -0.13 | -0.19 | 0.40 | 0.26 | 0.17 | -0.09 | -0.11 |
| **TNC** | 0.02 | 0.39 | -0.09 | 0.42 | 0.03 | -0.07 | -0.18 |
| **TNA** | 0.34 | 0.15 | -0.02 | 0.16 | 0.19 | -0.05 | -0.06 |
| **NUE** | 0.31 | -0.22 | 0.07 | -0.23 | 0.16 | 0.02 | 0.06 |
| **NUtE** | -0.03 | -0.39 | 0.09 | -0.41 | -0.04 | 0.07 | 0.17 |
| **Eigenvalue** | 6.49 | 3.08 | 2.81 | 2.39 | 2.18 | 1.56 | 1.30 |
| **Percentage of Variance (%)** | 25.94 | 12.33 | 11.25 | 9.56 | 8.73 | 6.24 | 5.20 |
| **Cumulative (%)** | 25.94 | 38.27 | 49.52 | 59.08 | 67.81 | 74.05 | 79.25 |

25 morphological traits with CV (**Supplementary Table 1**) were used for factor analysis using the principal component analysis (PCA) extraction method. For each trait, the largest variable loading score crossing the two components appears in bold. Principal components with eigenvalues > 1 are presented and considered significant.
